# Supplementary figures and images for: Estimating uncertainty in density surface models
Source: PeerJ. 2022 Aug 23;10:e13950. doi: 10.7717/peerj.13950 (PMC9415456; doi:10.7717/peerj.13950)

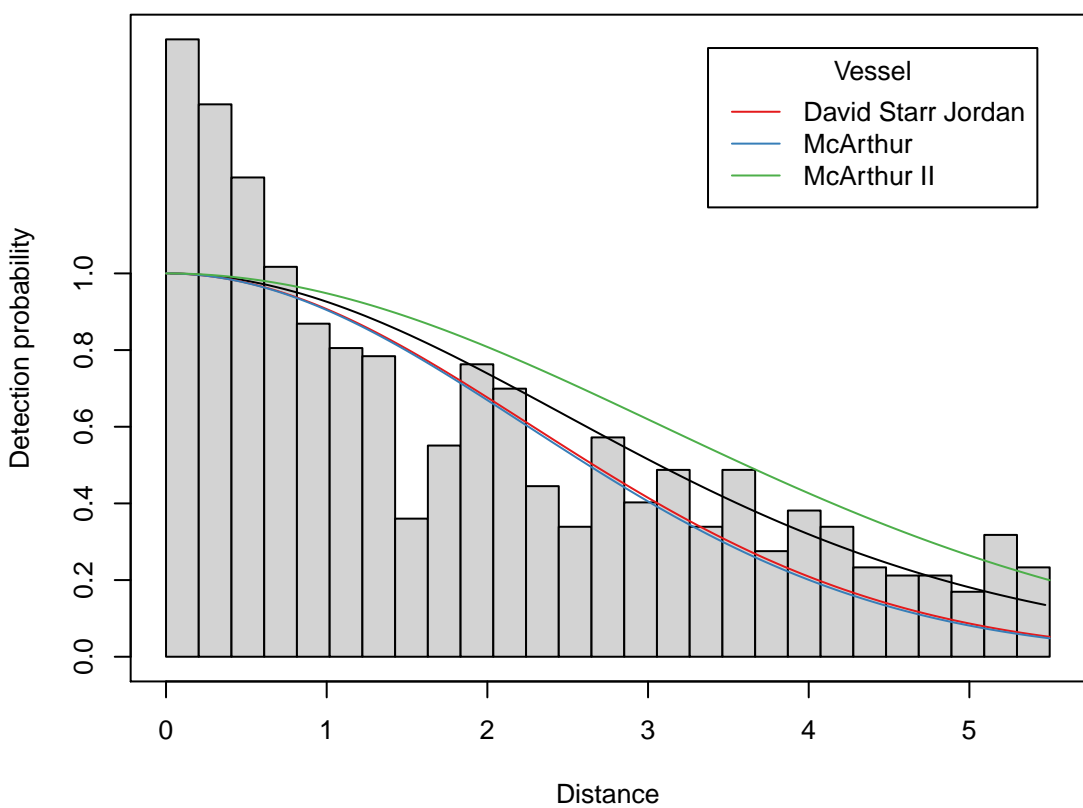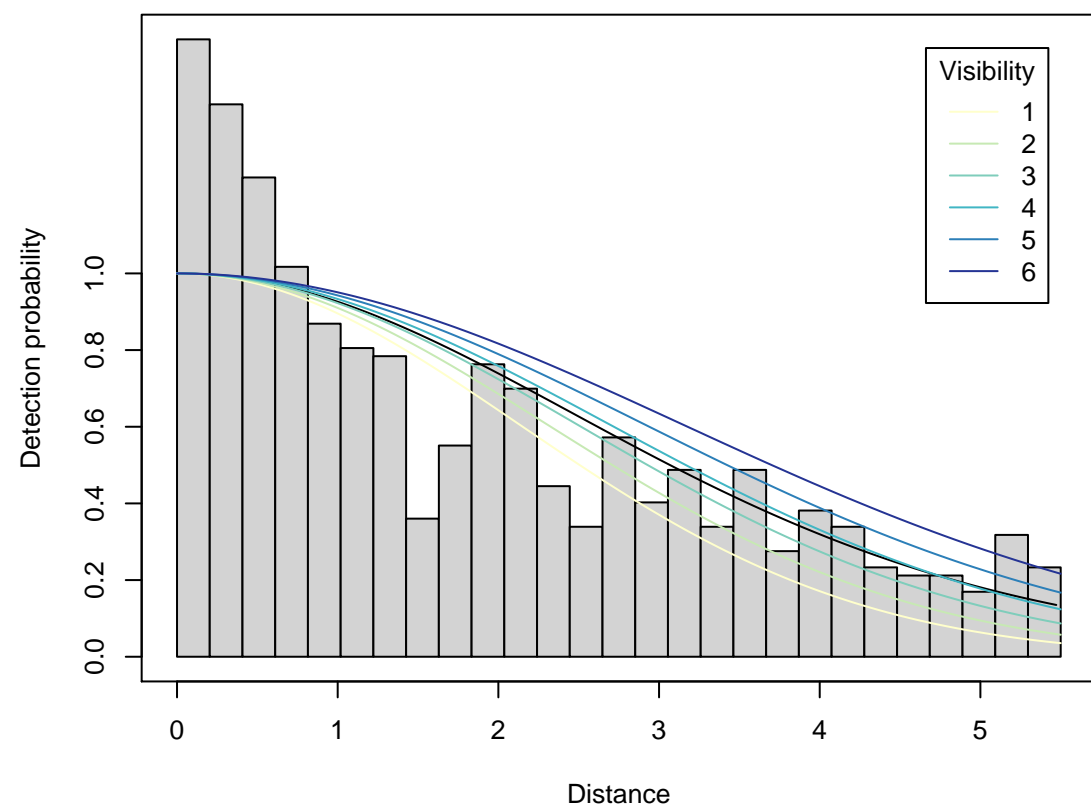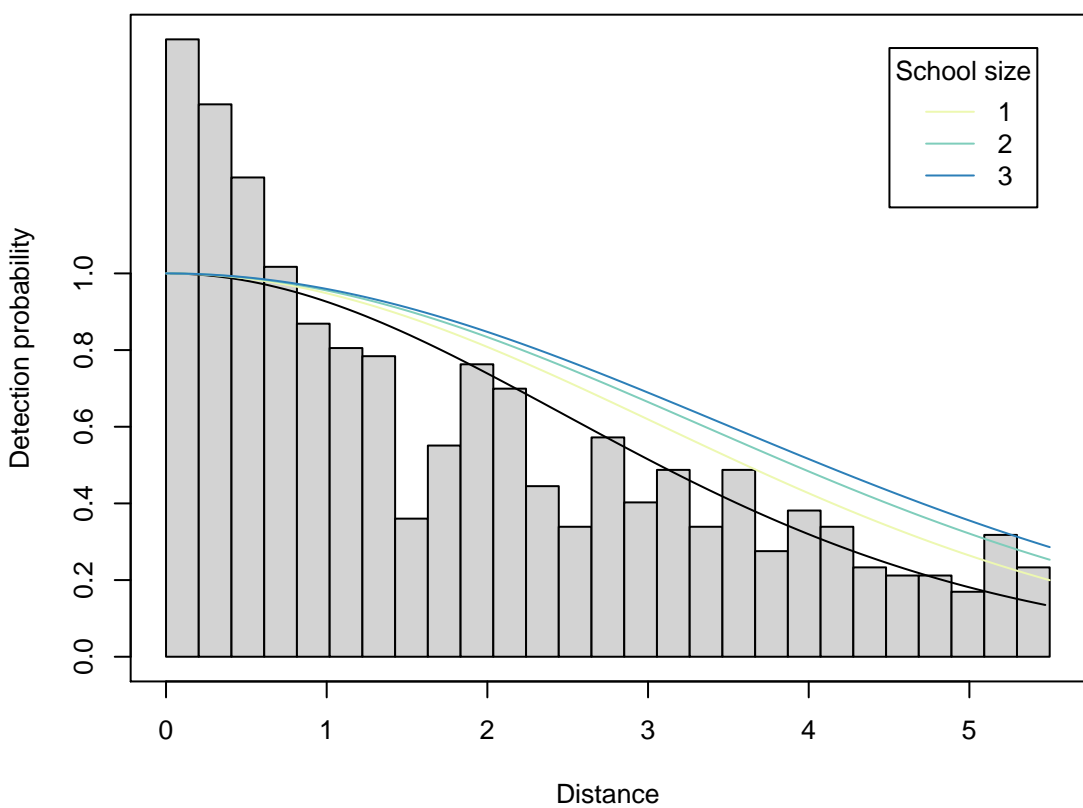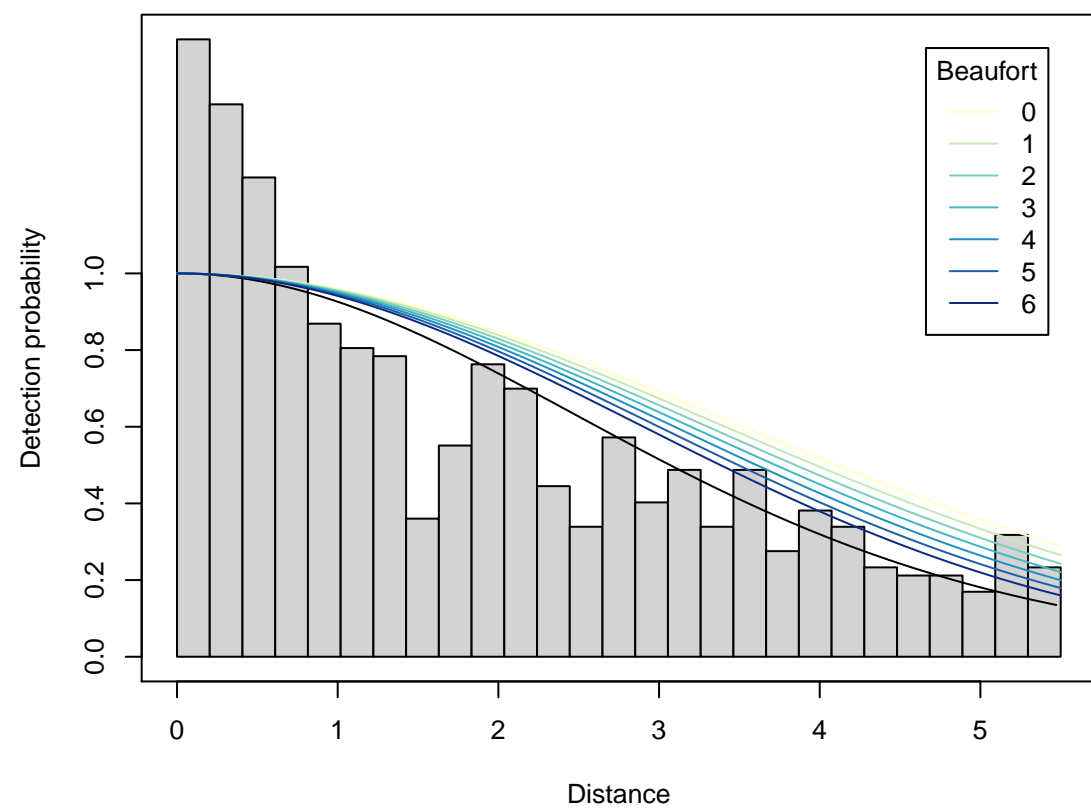

Supplement: Supplemental Information 1 — Black lines show average detection functions. Coloured lines give the detection function varying the given covariate with other covariates fixed. These fixed were vessel (segment mode) “McArthur II”, visibility (segment median) 5.67, school size 1, Beaufort (segment median) and species set to fin whale. [file peerj-10-13950-s001.pdf]

1996

2001

2005

2008

2014

Density

Standard error

Value

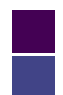

(0.0000, 0.0010]

(0.0010, 0.0023]

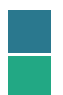

(0.0023, 0.0036]

(0.0036, 0.0085]

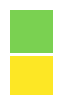

(0.0085, 0.0360]

(0.0360, 1.0000]

Supplement: Supplemental Information 2 — Top row: predicted yearly densities for fin whales in the California Current Ecosystem. Bottom row: yearly estimated standard errors for the predictions, using our procedure. Both measures are plotted on the same scale for easy comparison. Black dots give locations of observations of fin whales. [file peerj-10-13950-s002.pdf]

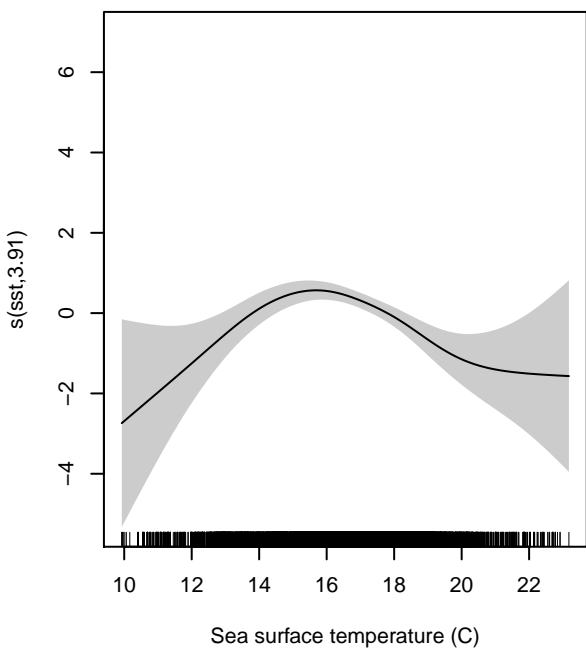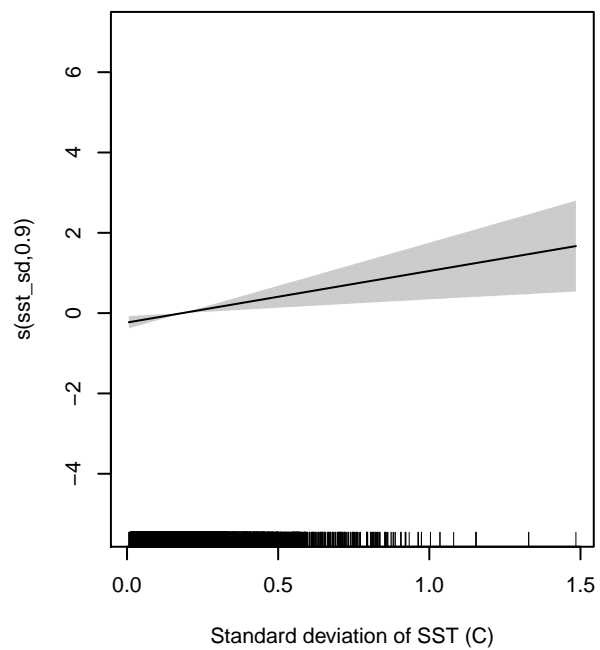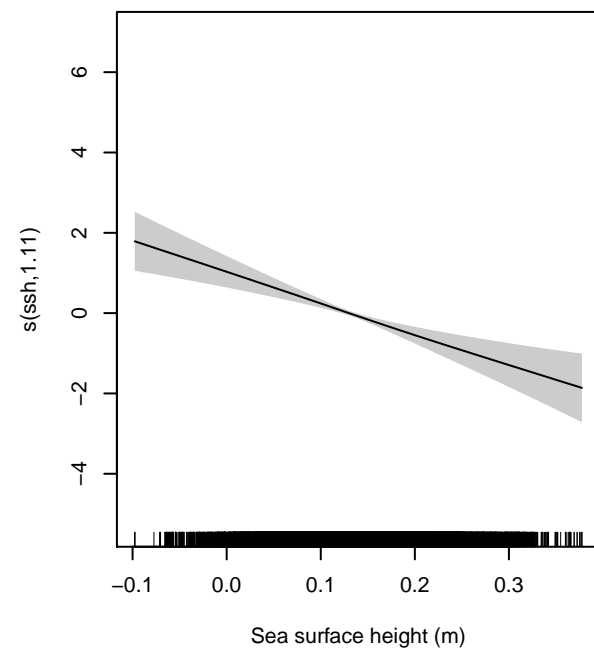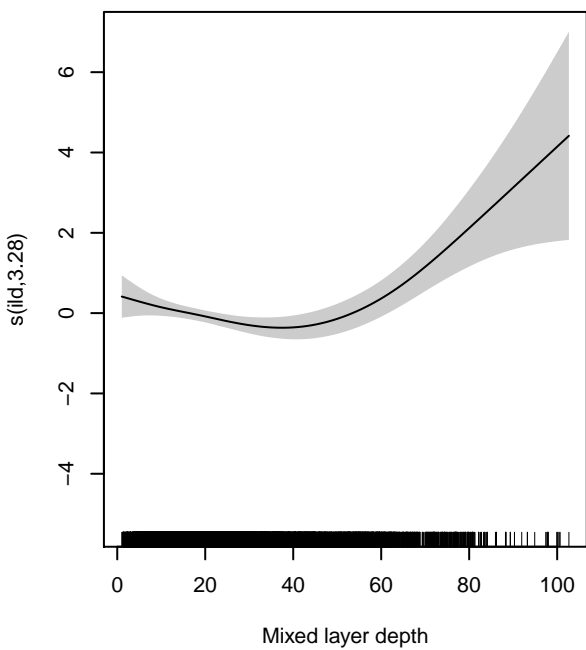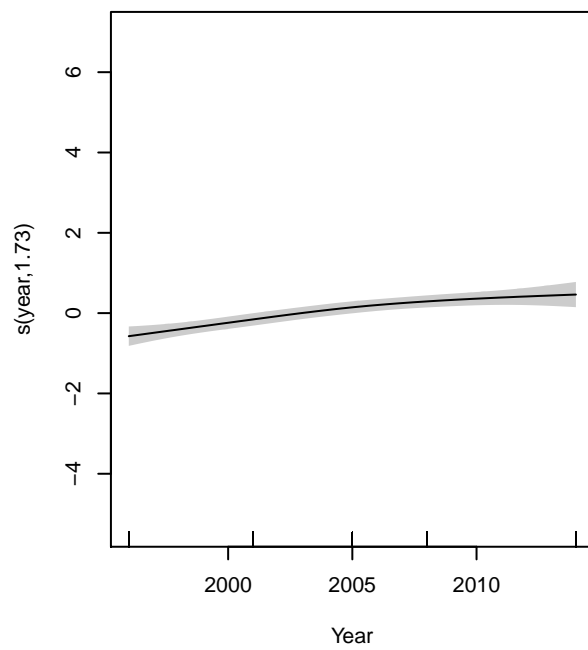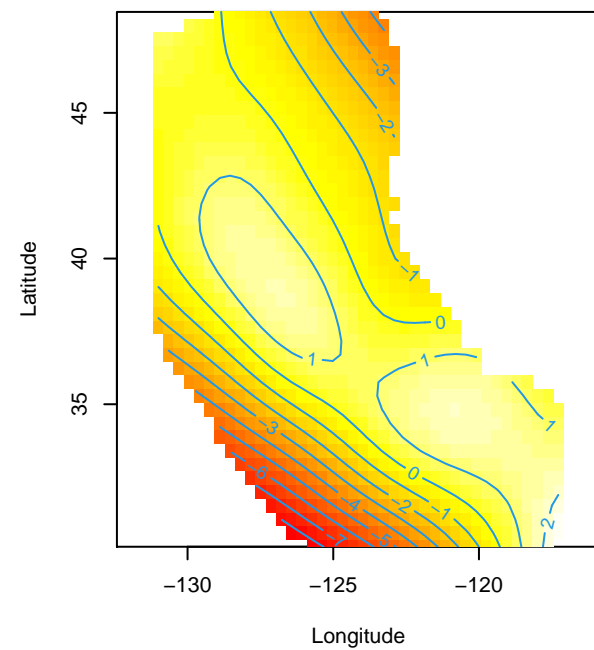

Supplement: Supplemental Information 3 — Top row and bottom row left and centre show univariate effects of the covariates, grey bands give the mean estimates +/ − 2 standard errors. The effective degrees of freedom are given in brackets on the vertical axes and rug plots show data locations. Bottom right plot shows the two dimensional tensor product spatial smooth with 15.41 effective degrees of freedom. [file peerj-10-13950-s003.pdf]

Mixed layer depth

Sea surface height

Sea surface temperature

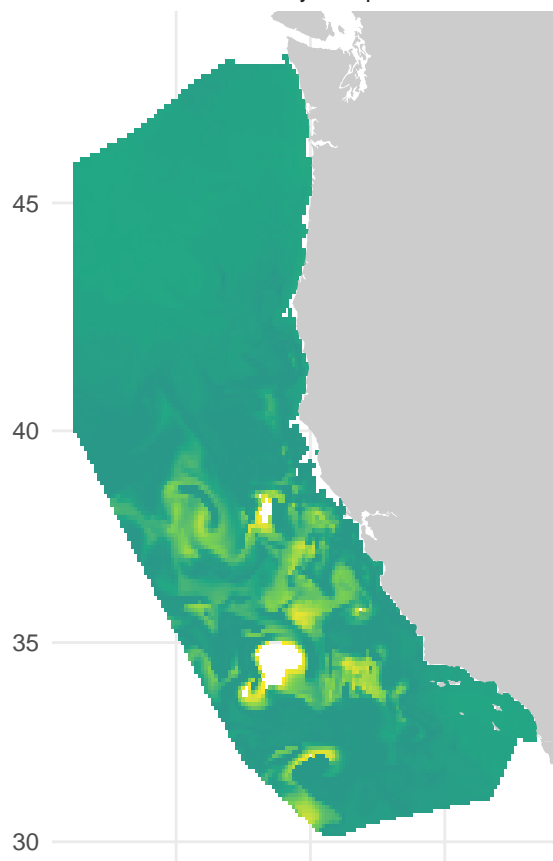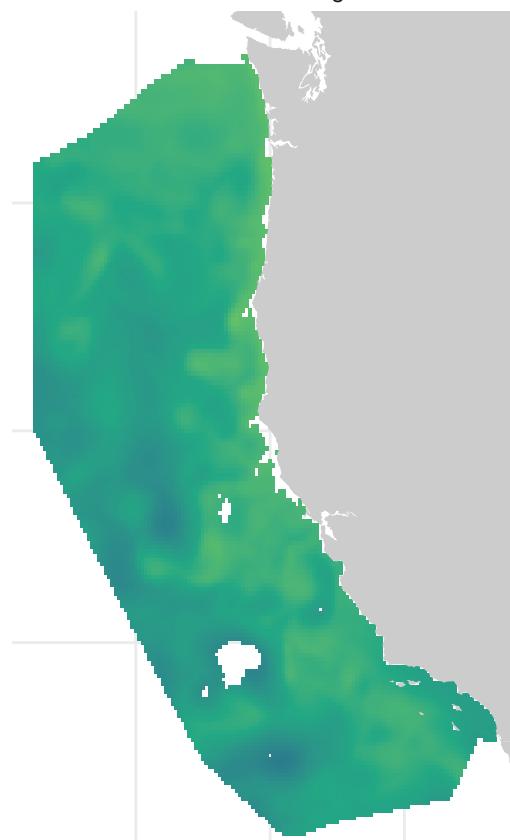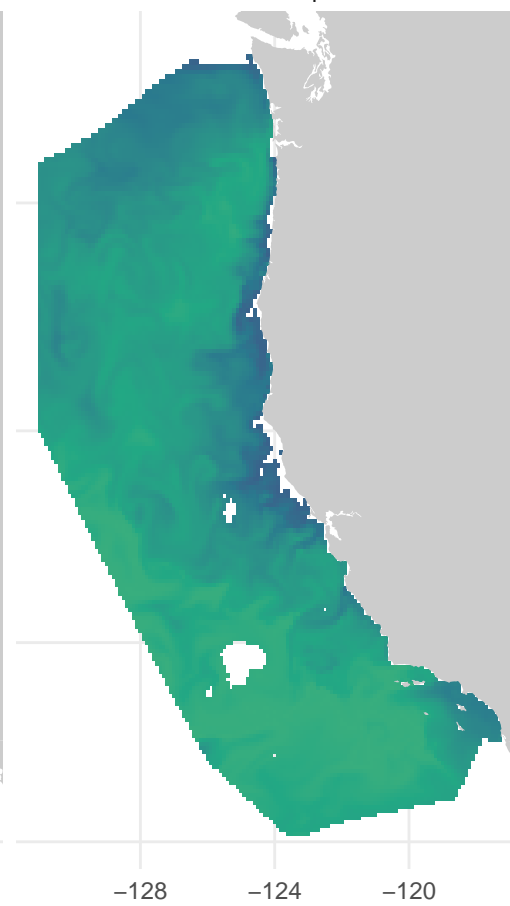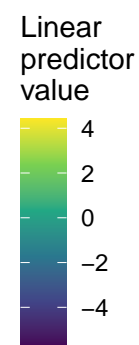

Space

Standard deviation of SST

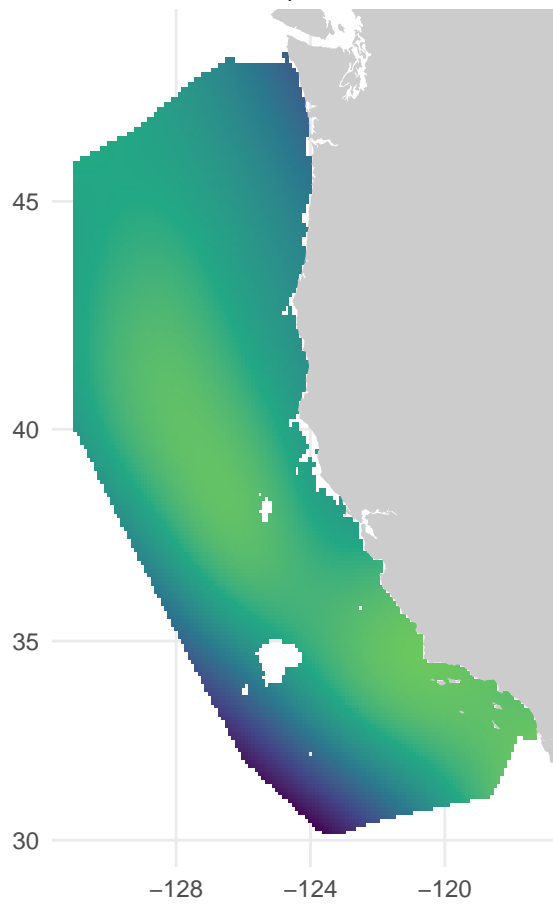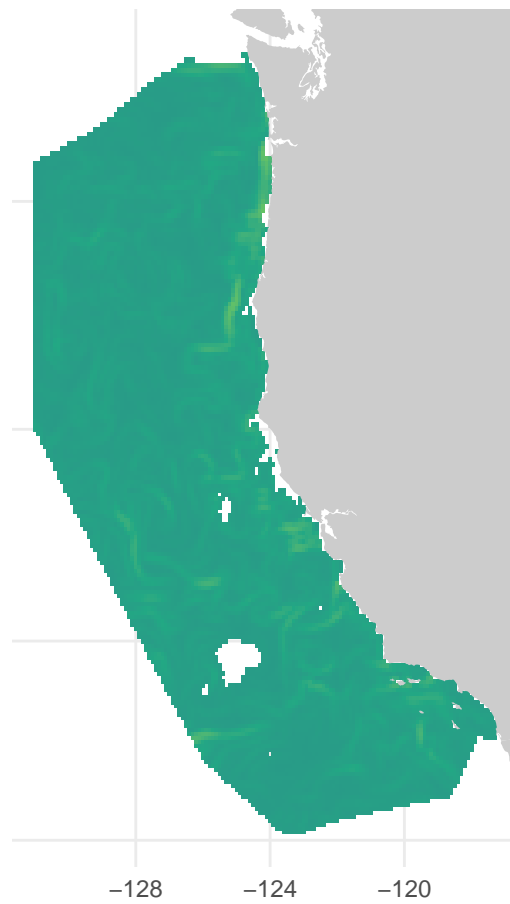

Supplement: Supplemental Information 4 — This was when there were large observed values of mixed layer depth, causing the large uncertainty in Figure 3. Plots are directly comparable in terms of their influence and were generated using the function plot_pred_by_term in the R package dsm. [file peerj-10-13950-s004.pdf]

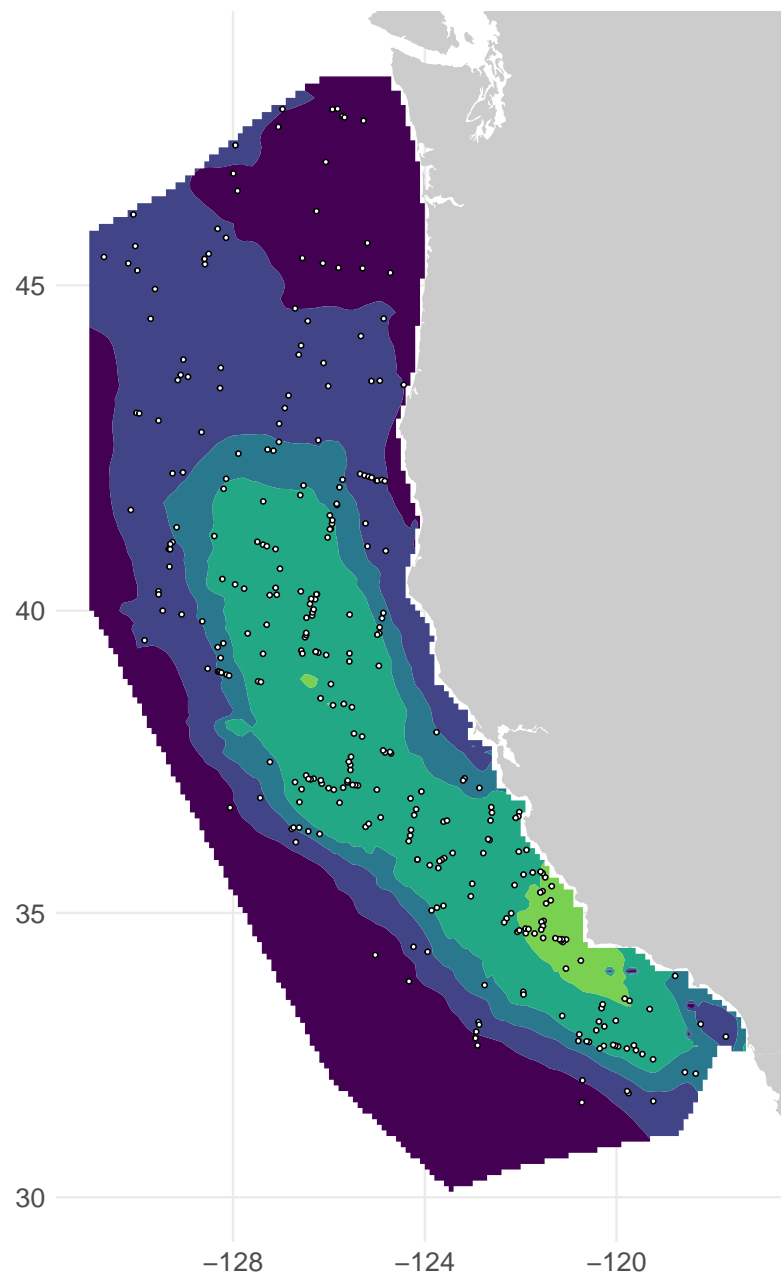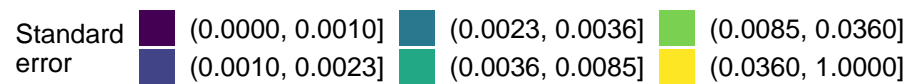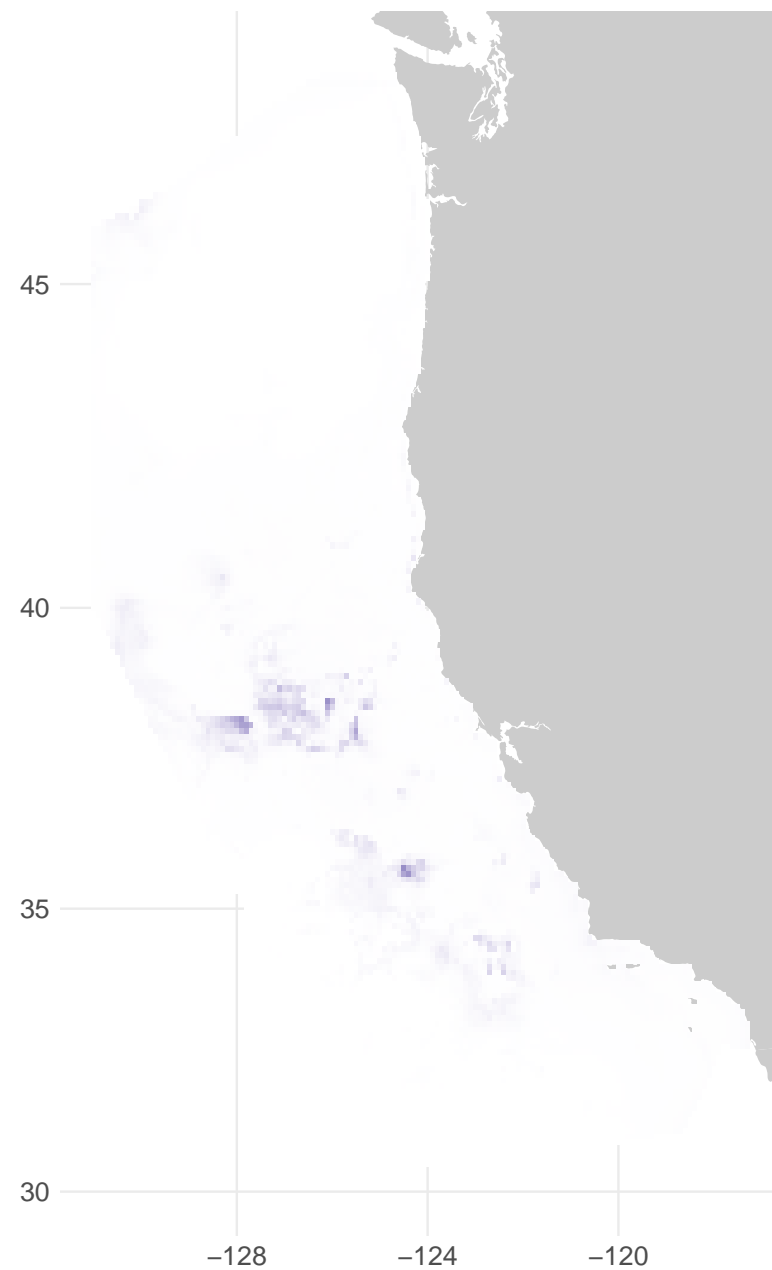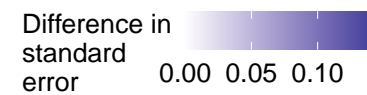

Supplement: Supplemental Information 5 — Left: Estimated standard error for the predictions, using only environmental uncertainty. Dots give locations of observations of fin whales. Right: differences between the full uncertainty in Figure 2 (right panel) and the left plot here. Note that as expected the uncertainty is always larger for the full uncertainty procedure. [file peerj-10-13950-s005.pdf]

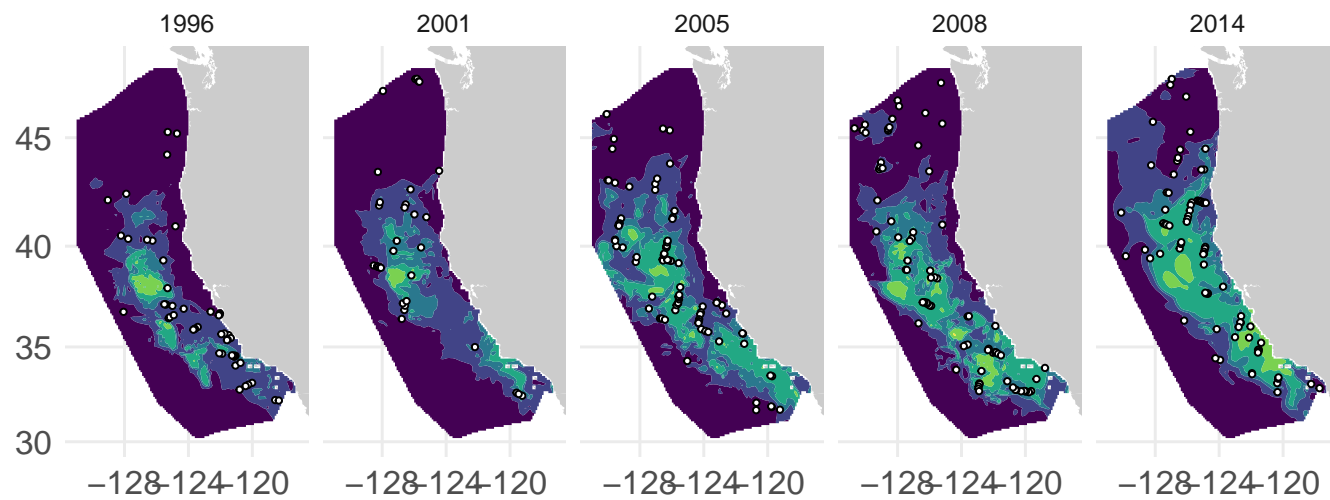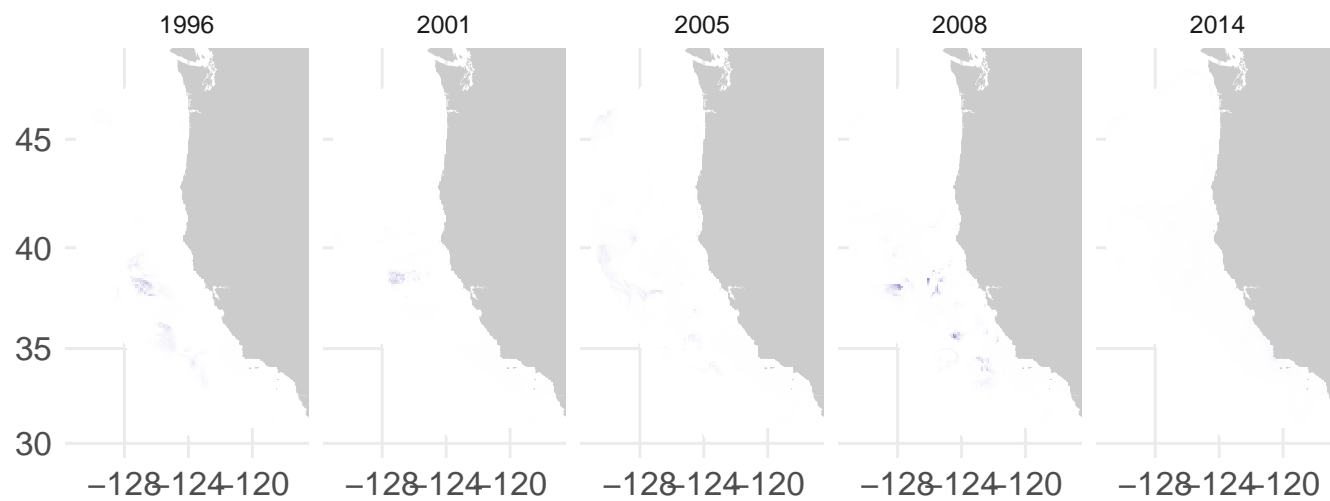

Supplement: Supplemental Information 6 — Top row: yearly estimated standard errors for the predictions, using only environmental uncertainty. Dots give locations of observations of fin whales. Bottom row: differences between the full uncertainty in Figure S2 and the top row here. Note that as expected the uncertainty is always larger for the full uncertainty procedure. [file peerj-10-13950-s006.pdf]
