# Supplementary material for: Estimating uncertainty in density surface models
Source: PeerJ. 2022 Aug 23;10:e13950. doi: 10.7717/peerj.13950 (PMC9415456; doi:10.7717/peerj.13950)
Supplement: Appendix S1 [file peerj-10-13950-s007.pdf]

Appendix A: Making predictions and estimating variance at and over multiple time points

David L Miller, Elizabeth A Becker, Karin A Forney,
Jason J Roberts, Ana Cañadas and Robert S Schick

June 21, 2022

Introduction

When making predictions of animal abundance from density surface models, we are interested in two situations: summaries for each prediction cell to create maps of uncertainty and summaries of total abundance (sums of the cells) over subsets of the prediction grid to obtain time series of abundance. This note investigates what different averages mean for predictions from spatial models based on generalized additive models (such as density surface models) when predictions are made at multiple time points and summaries of those predictions are required. Both simulation and analytical approaches are covered.

Setup

We begin by thinking about a spatial model based on a generalized additive model with an offset based on effort and detectability as in equation (1) in the main paper. In this case we have some models of the form:

$$\mathbb{E}(n_j) = A_j \hat{p}_j \exp\left(\beta_0 + \sum_k f_k(x_{kj})\right), \tag{1}$$

where n_j is the number of individuals (or groups) observed in segment j (of which there are J), which has some environmental covariates $x_{\cdot j}$ which are modelled by smooth functions f_k and some intercept β_0. Each segment is of size A_j and has associated detection probability $\hat{p}_j$ (as in the main article, we can extend this to include other components but stick with just detection probability for simplicity of presentation here).

We make predictions at each grid cell by plugging-in values of the covariates to the following equation:

$$\hat{n}_r = A_r \exp\left(\hat{\beta}_0 + \sum_k \hat{f}_k(x_{kr})\right),$$

where the predictions $\hat{n}_r$ are indexed by r, of size A_r (there are R total cells). Hats indicate estimated quantities. Noting that we represent the f_k as basis expansions, we can re-write the expression inside the bracket as a matrix-vector product:

$$\hat{n}_r = A_r \exp\left(\bar{\mathbf{X}}_r \hat{\boldsymbol{\beta}}\right), \tag{2}$$

where $\bar{\mathbf{X}}_r$ is a single row (corresponding to prediction cell r) of some prediction matrix, $\bar{\mathbf{X}}$ (Wood, 2017, Section 6.10). As well as being interested in $\hat{n}_r$, we are also interested in aggregates like $\hat{N} = \sum_{r=1}^{R} \hat{n}_r$ i.e., the abundance in a given area. Here we are interested in how to calculate uncertainties for $\hat{n}_r$ and $\hat{N}$ when we may have multiple estimates because of temporal variation in the x_{kr}.

Prediction targets

When all covariates in our model are static in time (e.g., bathymetry, altitude etc) things are straightforward, but it is often the case that environmental covariates are dynamic and therefore implicitly temporal (e.g., sea surface temperature, measures of prey distribution, etc). In which case, we make predictions for given time *slice,* e.g.,

"Friday 7 May 2021" or "week 16 of 2010", "an average day in July over the last 10 years" (the latter being an example of a "climatology"). We can make separate predictions for each of these time slices but we may also want to average them (for a given *period*), e.g., "average day in June in 2014 from predictions of each day in June in 2014". Note that below we generally assume that the slices are representative of the periods that contain them (that they are not just extrema).

Next we enumerate the options that we are interested in. Each is informally titled, followed by mathematical definition of an appropriate abundance estimator and a description of the corresponding variance. We describe how to calculate these in the next section. Note that in all of these cases we are making assumptions about the "representativeness" of the covariates we use to illustrate the conditions in our prediction area during the time of interest.

1. **Maps of abundance at a given slice.** Abundance for each prediction cell in a given time slice: $\{\hat{n}_r; r = 1, \ldots, R\}$. $\text{Var}(\hat{n}_r)$ gives uncertainty from model parameters for cell r given the environmental covariates. This is the "simple" case where we only have one prediction grid at a single slice and want to know about the resulting map and its uncertainty.

2. **Total abundance at a given slice.** Sum of 1., over all cells in this time slice: $\hat{N} = \sum_r \hat{n}_r$. $\text{Var}(\hat{N})$ gives uncertainty from the model parameters given covariates but including covariances between the cells. This summary of 1. is another simple case where we only have one time slice.

3. **Overall average map of abundance in a given period.** Average cell values in this period, made up of slices $t = 1, \ldots, T$: $\left\{\bar{n}_r = \frac{1}{T}\sum_t \hat{n}_{rt}; r = 1, \ldots, R\right\}$. $\text{Var}(\bar{n}_r)$ takes into account the variance both within and between the slices. Here we want to summarize over multiple environmental conditions (covariate grids) to get an idea of an average abundance map over some time period (e.g., "any day in 2014" or "any day in June in the last 10 years").

4. **Overall average total abundance in a given period.** Average of T estimates of 2., or the sum of $\hat{n}_{rt}$ over r, then averaged over T: $\bar{N} = \frac{1}{T}\sum_t \hat{N}_t = \frac{1}{T}\sum_t \sum_r \hat{n}_{rt}$. This summary of 3. gives us a measure of the abundance on a given random slice in a given period.

5. **Average map of abundance in a given period from summaries.** Summaries are required at some grouping of t (say, G groups $\{\tau_g; g = 1, \ldots, G\}$ where τ_g is the set of time indices for group g and $\sum_g |\tau_g| = T$ if we denote the number of elements in group g as $|\tau_g|$), before being averaged at the higher level: $\left\{\bar{n}_r^G = \frac{1}{G}\sum_g \frac{1}{|\tau_g|}\sum_{t\in\tau_g} \hat{n}_{rt}; r = 1, \ldots, R\right\}$. This allows us to make predictions at the daily level, summarize them at the month then calculate what an average month looks like over multiple years (rather than 3., which gives us a random day in that month). $\text{Var}(\bar{n}_r^G)$ also takes into account the variance both within and between periods rather than within and between slices.

6. **Average total abundance in a given period from summaries.** Summaries are required before calculating the total abundance. Again grouping at some lower level before averaging at the higher level but in this case for total abundance: $\bar{N}^G = \frac{1}{G}\sum_g \frac{1}{|\tau_g|}\hat{N}_g = \frac{1}{G}\sum_g \frac{1}{|\tau_g|}\sum_{t\in\tau_g}\hat{N}_t = \frac{1}{G}\sum_g \frac{1}{|\tau_g|}\sum_{t\in\tau_g}\sum_r \hat{n}_{rt}$. $\text{Var}(\bar{N}^G)$ gives our uncertainty in a total abundance estimate, taking into account variance within and between the total abundances per time period rather than per slice.

Obtaining point estimates of predictions for cases 1-6 is "easy", but what the variance estimates are less clear. We next look into general tactics for estimating variance in these cases, then come back to 1-6 and address them each in detail.

Tools for uncertainty estimation

We have two approaches to deal with uncertainty estimation from these kinds of models: simulation and analytic. Here we give a brief overview of these two methods in general before applying them to the above situations in the next section. Note that we assume here that uncertainty in $\hat{p}_j$ in (1) has be propagated via e.g, Bravington et al. (2021).

Simulation

The simulation approach is based on the idea that the $\hat{\boldsymbol{\beta}}$ estimated in our model has a (posterior) distribution that we can sample from. If we think of models fitted by REML as empirical Bayes estimates then we might call this *posterior sampling*, else we can call it a *parametric bootstrap*. Either way, we sample from the distribution of $\hat{\boldsymbol{\beta}}$, plug those samples into (2) and obtain multiple realisations of our predictions, based on the uncertainty of our model.

The posterior for $\boldsymbol{\beta}$ given smoothing parameters $\boldsymbol{\lambda}$ is *approximately* distributed as $\boldsymbol{\beta}|\boldsymbol{\lambda} \sim N(\hat{\boldsymbol{\beta}}, \mathbf{V}_{\hat{\boldsymbol{\beta}}})$, where as in the main article smoothing parameter uncertainty can also be included (but again we keep things simple here for presentational purposes). The following algorithm then can be used:

1. For $b = 1, \ldots, B$:
   (a) Simulate model coefficients from the fitted model, to obtain $\hat{\boldsymbol{\beta}}_b$ (this can be done directly from $N(\hat{\boldsymbol{\beta}}, \mathbf{V}_{\hat{\boldsymbol{\beta}}})$, using importance sampling or Metropolis-Hastings sampling).
   (b) Calculate per-cell predictions for this realisation by plugging $\hat{\boldsymbol{\beta}}_b$ into 2.
   (c) Calculate an appropriate summary statistic of the per-cell predictions (e.g., sum for total abundance).
2. Calculate the empirical variance or percentile intervals of the results from 1(c) to obtain requisite quantities.

The overarching idea here is that we are able to create "possible" predictions conditional on the model being correct. Our concern below will be how to calculate appropriate summaries at 1(c) and their variances at 2.

Analytic

The analytic approach is based on standard linear modelling theory. We can obtain $\mathbf{V}_{\hat{\boldsymbol{\beta}}}$, the covariance matrix for the model parameters (Wood, 2017, Sections 5.8 & 6.9.3). Using the delta method to move from uncertainty about $\hat{\boldsymbol{\beta}}$ to uncertainty about $\hat{n}_r$ or $\hat{N}$. For example, if we wanted to calculate the variance of a total abundance estimate $\hat{N}$, we use:

$$\text{Var}(\hat{N}) = \left(\mathbf{A} \frac{\partial \exp \bar{\mathbf{X}}\boldsymbol{\beta}}{\partial \boldsymbol{\beta}} \bigg|_{\boldsymbol{\beta}=\hat{\boldsymbol{\beta}}} \right) \boldsymbol{V}_{\hat{\boldsymbol{\beta}}} \left(\mathbf{A} \frac{\partial \exp \bar{\mathbf{X}}\boldsymbol{\beta}}{\partial \boldsymbol{\beta}} \bigg|_{\boldsymbol{\beta}=\hat{\boldsymbol{\beta}}} \right)^{\mathsf{T}}, \tag{3}$$

where $\mathbf{A}$ is a row vector of the A_rs. Derivatives are evaluated at the estimated values of the model parameters. The terms in brackets can be though of as re-projecting the uncertainty from the $\hat{\boldsymbol{\beta}}$s into the space of the predicted abundance estimate.

Our secondary tool for the analytic case is an identity which we can use to find total variance given we have summaries. First let us imagine the case in which we have a random variable X that we measure at $t = 1, \ldots, T$ and denote x_t (standing-in for a single cell or a total abundance estimate). If we have have a measurement at each t then we can simply compute the empirical variance of the x_ts to get an estimate of the variance of X. However, the more complicated case is where we partition the x_ts into G groups (for example, if t are days but we only have monthly averages, $G = 12$). In that case, we no longer can access the individual x_t, just $\bar{x}_g$ which are averages of the K (say) x_ts in partition G (assuming each group is of the same size, K, we have that $T = KG$). We also have $\text{Var}_g(x)$ the variance of the x_ts in partition g. We can then take the regular estimator of variance, $\text{Var}(x) = \frac{1}{T-1} \sum_{t=1}^{T} (x_t - \bar{x})^2$, and re-arrange as follows to get an expression in terms of $\text{Var}_g(x)$ and $\text{Var}(\bar{x}_g)$ (the empirical variance of the $\bar{x}_g$s).

So we start out with:

$$
\begin{aligned}
\mathrm{Var}(x) &= \frac{1}{T-1}\sum_{t=1}^{T}(x_t - \bar{x})^2\\
&= \frac{1}{KG-1}\sum_{g=1}^{G}\sum_{k=1}^{K}(x_{gk} - \bar{x})^2\\
&= \frac{1}{KG-1}\sum_{g=1}^{G}\sum_{k=1}^{K}(x_{gk} + \bar{x}_g - \bar{x}_g - \bar{x})^2\\
&= \frac{1}{KG-1}\sum_{g=1}^{G}\sum_{k=1}^{K}[(x_{gk} - \bar{x}_g) + (\bar{x}_g - \bar{x})]^2\\
&= \frac{1}{KG-1}\sum_{g=1}^{G}\sum_{k=1}^{K}(x_{gk} - \bar{x}_g)^2 + \cancelto{0}{2(x_{gk} - \bar{x}_g)(\bar{x}_g - \bar{x})} + (\bar{x}_g - \bar{x})^2\\
&= \frac{1}{KG-1}\sum_{g=1}^{G}\sum_{k=1}^{K}\left[(x_{gk} - \bar{x}_g)^2 + (\bar{x}_g - \bar{x})^2\right]\\
&= \frac{1}{KG-1}\left[\sum_{g=1}^{G}\sum_{k=1}^{K}(x_{gk} - \bar{x}_g)^2 + \sum_{g=1}^{G}\sum_{k=1}^{K}(\bar{x}_g - \bar{x})^2\right]
\end{aligned}
$$

The summation in the right-hand term in the above is $G-1$ times the variance of the $\bar{x}_g$s, $\mathrm{Var}(\bar{x}_g)$ (from the definition of the variance). We can apply the same logic to the left side too, and say that the inner summation is $K-1$ times the per group variance of the x_{gk}s, $\mathrm{Var}_g(x)$. So, we can write:

$$
\begin{aligned}
\mathrm{Var}(x) &= \frac{1}{KG-1}\left[\sum_{g=1}^{G}(K-1)\mathrm{Var}_g(x) + K(G-1)\mathrm{Var}(\bar{x}_g)\right] \qquad (4)\\
&= \frac{(K-1)}{KG-1}\sum_{g=1}^{G}\mathrm{Var}_g(x) + \frac{K(G-1)}{KG-1}\mathrm{Var}(\bar{x}_g).
\end{aligned}
$$

Translating this to the kind of temporal summaries we are interested in, $\mathrm{Var}_g(x)$ represents uncertainty from the model (from the $\hat{\boldsymbol{\beta}}$s), and $\mathrm{Var}(\bar{x}_g)$ is a measure of the between-time-slice uncertainty from the estimates.

In the simple case of a random variable above, we know what K should be. This is less clear when we use (3) to obtain $\mathrm{Var}_g(x)$. By analogy to the simple case, K is the "number of samples used to calculate each $\mathrm{Var}_g(x)$", that is the number of samples used to fit the model from which we derive our predictions. So $K = J$, where J is the number of segments used to fit the model (see above). Since increasing K will improve our estimate of variance in the above, and increasing the number of segments will generally improve our estimate of variance, the analogue holds.

Estimating uncertainty in useful cases

Going back to our list above, how can we use the above tools to obtain useful results? Conceptually, the simulation approach can be easier to think about, as we are able to consider posterior samples as "possible universes" in which particular resulting populations occur. We can also think about how/whether we want to make sampling exchangeable with time slices of environmental covariates. Analytic results are more involved, as we need to work out the form of matrix operations, rather than the averaging required for the simulation approach. Below we look at the simulation-based approach first in each case and then use that to aid the construction of the analytic estimator.

Maps of abundance at a given slice

This is the simplest case, as described in Miller et al. (2013). We have one prediction grid and want to calculate the resulting map and its uncertainty. We use 2 to get $\hat{n}_r$ and calculate $\mathrm{Var}(\hat{n}_r)$ in one of the following two ways:

1. **Simulation**: Storing the $\hat{n}_r$ for each posterior sample $b = 1, \ldots, B$ then calculating the variance per cell over the B samples.

2. **Analytic**: Apply (3) to each cell:

$$\text{Var}(\hat{n}_r) = \left(A_r \frac{\partial \exp \bar{\mathbf{X}}_r \boldsymbol{\beta}}{\partial \boldsymbol{\beta}} \bigg|_{\boldsymbol{\beta}=\hat{\boldsymbol{\beta}}} \right) \boldsymbol{V}_{\hat{\boldsymbol{\beta}}} \left(A_r \frac{\partial \exp \bar{\mathbf{X}}_r \boldsymbol{\beta}}{\partial \boldsymbol{\beta}} \bigg|_{\boldsymbol{\beta}=\hat{\boldsymbol{\beta}}} \right)^{\mathsf{T}}, \tag{5}$$

for $r = 1, \ldots, R$.

Total abundance at a given slice

Now for a single time slice, sum cells in the map to obtain $\hat{N} = \sum_r \hat{n}_r$. Again, this is a case covered in Miller et al. (2013).

1. **Simulation**: At step 1(c), we store the $\hat{N}$ for each posterior sample $b = 1, \ldots, B$ then calculating the variance per over the B estimates of $\hat{N}$.

2. **Analytic**: $\text{Var}(\hat{N})$ is calculated as in (3).

Overall average map of abundance in a given period

Now we have the case where we obtain multiple estimates per prediction cell, based on different covariate values (slices) and want to get an average in a period (collection of T slices indexed by t). As noted above, we need to make an assumption about how representative both each slice is for that time and about how representative of the whole period a collection of slices is. For prediction cell r, $\bar{n}_r = \frac{1}{T} \sum_t \hat{n}_{rt}$.

1. **Simulation**: The simulation lets us think about this clearly: we want to allow for exchangeability between time and samples. The full set of TB possible values for a given cell (based on the values in the TB generated surfaces) can be considered different possible values taking into account both environmental variability and model uncertainty. So, we store $\hat{n}_{rtb}$ (the estimate for cell r at time t from posterior sample b) at step 1(c). We can then use the empirical variance to obtain :

$$\text{Var}(\bar{n}_r) = \frac{1}{TB - 1} \sum_{t,b} \left(\hat{n}_{rtb} - \bar{n}_r \right)^2. \tag{6}$$

2. **Analytic**: We want to find an equivalent analytic expression to (6): that is the variance around the average $\bar{n}_r$. We can obtain the variance per cell, per slice $\text{Var}_t(\hat{n}_{rt})$, using (5) (which only includes model uncertainty from $\hat{\boldsymbol{\beta}}$). The empirical variance of per-slice estimates is given by $\text{Var}\left(\hat{n}_t\right) = \frac{1}{T-1} \sum_t \left(\hat{n}_{rt} - \bar{n}_r\right)^2$ (which does not include any model uncertainty from $\hat{\boldsymbol{\beta}}$). Ideally we would like to have the T estimates be for each slice in the period we are interested in, at a representative temporal resolution (e.g., for a average over a month, we might ideally like to have daily slices but in practice only have 8-day averages). We can use (4) to write:

$$\text{Var}(\bar{n}_r) = \frac{(J-1)}{JT-1} \sum_{t=1}^{T} \text{Var}_t(\hat{n}_{rt}) + \frac{J(T-1)}{JT-1} \text{Var}(\hat{n}_{rt}). \tag{7}$$

Overall average total abundance in a given period

We can take the T maps implied by the predictions in the previous subsection and sum each to obtain estimates of abundance per slice, then average these for an overall average. The point estimate of abundance can therefore be calculated in terms of the estimates of abundance per time period ($\hat{N}_t$) or from the perspective of the cells, to obtain: $\bar{N} = \frac{1}{T} \sum_t \hat{N}_t = \frac{1}{T} \sum_t \sum_r \hat{n}_{rt}$.

1. **Simulation**: Via the same logic as in the previous situation, we now store $\hat{N}_{tb}$ at step 1(c), and summarize using $\text{Var}(\hat{N}) = \frac{1}{TB-1} \sum_{t,b} \left(\hat{N}_{tb} - \bar{N}\right)^2$.

2. **Analytic**: We can use the same approach as in (7), but this time we make summaries first, summing the per-cell observations (applying (3) to each of the T time slices to get $\text{Var}(\hat{N}_t)$ and calculating the empirical variance over the estimates $\hat{N}_t$ to obtain$\text{Var}_t\left(\hat{N}_t\right)$). We then get the following formula:

$$\text{Var}(\bar{N}) = \left[\frac{J-1}{TJ-1}\sum_{t=1}^{T}\text{Var}_t(\hat{N}_t)\right] + \left[\frac{J(T-1)}{TJ-1}\text{Var}\left(\hat{N}_t\right)\right]. \tag{8}$$

Here K has the same meaning as in (7).

Average map of abundance in a given period from summaries

Our final two prediction targets require summarization before making a final estimate. We take our per-cell per-time slice estimates, $\hat{n}_{rt}$, within some grouping and average those first, then take the overall and then take a final mean over the G groups we created: $\bar{n}_r^G = \frac{1}{G}\sum_g \frac{1}{|\tau_g|}\sum_{t\in\tau_g}\hat{n}_{rt}$. As a concrete example, if we have daily estimates and want a seasonal summary map but assuming an average day in each month, we would have t indexing the T days, G months index by g each of length τ_g days. So $\text{Var}(\bar{n}_r^G)$ gives the variance of an average day in month g (for cell r).

1. **Simulation**: At step 1(c), we calculate within each grouping for each posterior sample per cell, so we calculate $\bar{n}_{rb}^g = \frac{1}{|\tau_g|}\sum_{t\in\tau_g}\hat{n}_{rtb}$ (note the b subscript remains so model uncertainty is retained). Then when we come to summarize we calculate the empirical variance over the groups and posterior samples $\bar{n}_{rb}^g$, $\text{Var}(\bar{n}_r^G) = \frac{1}{GB-1}\sum_{g,b}\left(\hat{n}_{rb}^g - \bar{n}_r^G\right)^2$.
2. **Analytic**: As above, we have variance per cell, per slice $\text{Var}(\hat{n}_{rt})$. We can obtain our estimator by using (4) twice: first at the "top level" to obtain an estimate over the G groups, then within each of the G groups. Starting with the former, we write:

$$\text{Var}(\bar{n}_r^G) = \frac{J-1}{GJ-1}\left[\sum_{g=1}^{G}\text{Var}_g(\bar{n}_r^g) + \frac{J(G-1)}{J-1}\text{Var}\left(\bar{n}_r^g\right)\right], \tag{9}$$

where $\text{Var}\left(\bar{n}_r^g\right)$ gives the empirical between-group variance ($\text{Var}(\bar{n}_r^g) = \frac{1}{|\tau_g|}\sum_{t\in\tau_g}\left(\hat{n}_r^g - \bar{n}_r^g\right)^2$). We apply (4) again to obtain a usable expression for $\text{Var}_g(\bar{n}_r^g)$, we can then write:

$$\text{Var}_g(\bar{n}_r^g) = \frac{(J-1)}{J\,|\tau_g|-1}\sum_{t\in\tau_g}\text{Var}_t(\hat{n}_{rt}) + \frac{J(|\tau_g|-1)}{J\,|\tau_g|-1}\text{Var}(\hat{n}_{rt})_{t\in\tau_g}, \tag{10}$$

where $\text{Var}(\hat{n}_t)_{t\in\tau_g}$ is the empirical variance of those estimates in group g and $\text{Var}_t(\hat{n}_{rt})$ is calculated from the delta method (3) as above. So substituting (10) into (9) we obtain:

$$\text{Var}(\bar{n}_r^G) = \frac{J-1}{GJ-1}\left\{\sum_{g=1}^{G}\left[\frac{(J-1)}{J\,|\tau_g|-1}\sum_{t\in\tau_g}\text{Var}_t(\hat{n}_{rt}) + \frac{J(|\tau_g|-1)}{J\,|\tau_g|-1}\text{Var}(\hat{n}_{rt})_{t\in\tau_g}\right] + \frac{J(G-1)}{J-1}\text{Var}\left(\bar{n}_r^g\right)\right\}. \tag{11}$$

Average total abundance in a given period from summaries

Finally we have the situation where we have the same grouping setup as the last situation, but now we want total abundance estimates, rather than maps. Our point estimate is now $\bar{N}^G = \frac{1}{G}\sum_g \frac{1}{\tau_g}\hat{N}_g = \frac{1}{G}\sum_g \frac{1}{\tau_g}\sum_{t\in\tau_g}\hat{N}_t = \frac{1}{G}\sum_g \frac{1}{\tau_g}\sum_{t\in\tau_g}\sum_r \hat{n}_{rt}$. $\text{Var}(\bar{N}^G)$ gives our uncertainty in a total abundance estimate, taking into account variance within and between the total abundances per time period rather than per slice.

1. **Simulation**: At step 1(c), we calculate within each grouping for each posterior sample per cell, so we calculate $\bar{N}_b^G = \frac{1}{G}\sum_g \frac{1}{\tau_g}\sum_{t\in\tau_g}\sum_r \hat{N}_{tb}$ (note the b subscript remains so model uncertainty is retained). Then when we come to summarize we calculate the empirical variance over the groups and posterior samples $\bar{N}_b^G$, $\text{Var}(\bar{N}^G) = \frac{1}{GB-1}\sum_{g,b}\left(\hat{N}_b^g - \bar{N}^G\right)^2$.

2. **Analytic**: We can adapt (11) to the case of $\bar{N}^G$:

$$\text{Var}(\bar{N}^G) = \frac{J-1}{GJ-1}\left\{\sum_{g=1}^{G}\left[\frac{(J-1)}{J\left|\tau_g\right|-1}\sum_{t\in\tau_g}\text{Var}_t(\hat{N}_t) + \frac{J(\left|\tau_g\right|-1)}{J\left|\tau_g\right|-1}\text{Var}(\hat{N}_t)_{t\in\tau_g}\right] + \frac{J(G-1)}{J-1}\text{Var}\left(\bar{N}^g\right)\right\}, \quad (12)$$

where quantities are analogous to those above.

Discussion

Here we have developed estimators for some common situations when attempting to calculate abundance estimates for populations when model include temporally-varying covariates. We tend to prefer the simulation-based approaches on a conceptual level, we also note that in our experience these estimators are often easier to understand for a non/less mathematically-inclined audience. This approach is not without its problems though, ensuring that sampling of $\hat{\boldsymbol{\beta}}$ is correct can be tricky, and we have found that the multivariate normal assumption does not hold in many situations, prompting the use of the more expensive Metropolis-Hastings algorithm. This problem of poor sampling is often revealed by the presence of extreme abundance predictions in the set of simulations, which are more likely to occur when fitted smooth terms, as plotted on the log scale, exhibit strongly widening intervals above zero, as for mixed layer depth in Figure S3, especially when predictions are made beyond the sampled ranges of such covariates. Another reason to prefer the simulation-based approach is that percentile intervals can be calculated easily, this is more tricky in the analytic case as the usual assumption of abundance being log-normally distributed does not necessarily hold.

References

M. V. Bravington, D. L. Miller, and S. L. Hedley. Variance Propagation for Density Surface Models. *Journal of Agricultural, Biological and Environmental Statistics*, Feb. 2021. ISSN 1085-7117, 1537-2693.

D. L. Miller, M. L. Burt, E. A. Rexstad, and L. Thomas. Spatial models for distance sampling data: recent developments and future directions. *Methods in Ecology and Evolution*, 4(11):1001–1010, 2013.

S. N. Wood. *Generalized Additive Models. An Introduction with R.* Texts in Statistical Science. CRC Press, 2nd edition, 2017.
